# Patient-Related Factors Influencing Satisfaction in the Patient-Doctor Encounters at the General Outpatient Clinic of the University of Calabar Teaching Hospital, Calabar, Nigeria

**DOI:** 10.1155/2012/517027

**Published:** 2012-05-20

**Authors:** Ndifreke E. Udonwa, Udoezuo K. Ogbonna

**Affiliations:** Department of Family Medicine, University of Calabar Teaching Hospital, P.O. Box 147, Calabar 540001, Cross Rivers State, Nigeria

## Abstract

Medical consultation is at the centre of clinical practice. Satisfaction of a patient with this process is a major determinant of the clinical outcome. This study sought to determine the proportion of patients who were satisfied with their doctor-patient encounter and the patient-related factors that affected patients' satisfaction with the consultation process. A clinic-based, cross-sectional study using a modified version of the General Practice Assessment Questionnaire (GPAQ), which employed a systematic sampling technique, was used. The questionnaires were administered on 430 patients within the ages of 18 years and 65 years. Among the 430 subjects within the ages of 18 years and 65 years studied, 200 (46.5%) were males and 230 (53.5%) were females. Only 59.3% were satisfied with their patient-doctor encounter. The patient's perception of time spent in the consultation, illness understanding after the visit, ability to cope with the illness after the visit, and ability to maintain health after visit were the only factors that affected patient's satisfaction with the consultation. In our environment, nonsatisfaction with the patient-doctor encounter is high. Only few factors considered to encourage a patients satisfaction at primary care consultation contributed to end-of-consultation satisfaction. This calls for refocusing so as to improve the overall patient care in our cultural context and meet the patient needs in our environment.

## 1. Introduction

There is an increasing interest in the study of the consultation process and patients' satisfaction with it [1]. The core activity in primary care is the consultation irrespective of whether patients consult for cure, services, counseling, prevention, or care. A widely accepted model views the consultation as a dialogue involving elements of negotiation to create a common reality to which agenda setting is paramount [2].

In the medical consultation the doctor and patient meet on common grounds with tolerance for each other's rights. This consultation by necessity requires a doctor who is expected to possess the requisite knowledge which will be useful in solving the problems the patient presents with the assumption that the doctor will act in the best interest of the patient. Guided by rules of professional conduct, objectivity, and being emotionally detached the doctor is guaranteed the right to examine the patient physically and to enquire into intimate areas of the patient's physical and emotional life.

During the consultation, the reason for attendance is defined and an appropriate action is chosen. This process aims at achieving a shared understanding, involving the patient in management and using time and resources appropriately.

Physicians have been noted to have fixed ideas about what is best for a patient, and this inflexibility leaves little room for negotiation. Quite often animosity is expressed when the patient attempts to negotiate [3]. However, as demonstrated by a study in the Netherlands, interindividual and intra-individual variability does occur among physicians, who were noted to adjust their styles according to the situation [4].

This study sought to identify the factors contributing to patients' satisfaction, shed more light on the burden of patients' dissatisfaction with the consultation in our environment, and help devise strategies for practicing physicians to strive for an improvement in the overall patient care in our cultural context. The findings would provide some of the information needed to further fill the knowledge gap about our patient needs in our environment and highlight the need to teach the consultation process at both undergraduate and postgraduate medical training. This study comes from a background where patient awareness of their opportunities in the patient doctor encounter is still in its early days, and thus this study brings to the literature a unique perspective of the patients' views from this environment.

## 2. Methods

This study was conducted in the General Outpatient Clinic (GOPC) of the University of Calabar Teaching Hospital, (UCTH) Calabar. The University of Calabar Teaching Hospital is a tertiary hospital located within Calabar metropolis, which lies along latitude 4°, 58′ North of the Equator and longitude 8°20′ East of the Greenwich Meridian [5]. The UCTH Calabar has four sites for service provision: the St. Margaret's annex, Maternity annex, the permanent site, and the Comprehensive Health Centre (CHC) Okoyong. The General Outpatient Clinic (GOPC) is situated at the permanent site and has fourteen outpatient consulting rooms in which about 10–14 doctors (family physicians or resident doctors in family medicine) consult from 8 am–4 pm on a daily basis. Three consulting rooms are for consultants, three for doctors dedicated to the HIV clinic, one for consultancy patients, one for patients who are staff, and six consulting rooms are for outpatient consultation by other doctors. The department also had 16 nurses, 6 records' personnel, 5 orderlies, 3 counselors, and 5 administrative staff who usually assisted the doctors during the consultation.

All adults between the ages of 18 and 65 years who consented to participate in the study were recruited. Informed consent was obtained from the patients before they were given a questionnaire to complete. The patients were recruited using a systematic random sampling technique. The average attendance of patients at the UCTH GOPC in the three months preceding the study was 74 patients per day. The number of working days within the month was 22 days. Therefore, the number of patients estimated to attend the clinic was 74 × 22 = 1628. From the calculated sample size of 430 subjects, the sampling interval was calculated as 1628 ÷ 430 = 3.79 or 4. A sampling interval of 4 was used to systematically select subjects who were recruited to participate in the study. Every fourth patient was invited to participate in the study. The patients' attendance register for each day was used as the sampling frame from which patients were selected. The first subject was chosen randomly from this sampling frame, and subsequently every fourth patient was selected and invited to participate. If a selected subject did not meet the inclusion criteria or refused to participate in the study, the next patient was approached until the recommended sample size was recruited.

A self-administered, pretested questionnaire adapted from the General Practice Assessment Questionnaire [6] was completed by consenting patients after their consultation.

The General Practice Assessment Questionnaire was developed in the United Kingdom and used to study certain components of the consultation. The questionnaire consisted of 21 questions divided into five sections that investigated the proportion of patients that are satisfied with their patient-doctor encounter, which patient-factors are associated with patients' satisfaction or lack of satisfaction with the consultation.

Some of the questions had options from which the patient selected the response while others made room for a narrative response. Prior to the commencement of the consultation, all the patients waiting to be consulted were addressed on the possibility of being approached to join an ongoing study. This address was repeated several times during the course of a day's consultation. Selected patients on leaving the consulting rooms were approached by the trained assistants and requested to complete the questionnaire.

Data generated in the study was analyzed using the Epi Info software for analyzing medical data from the Centre for Disease Control, Atlanta, Georgia, USA [7]. The Chi square test was used to test for significance.

 Privacy of the patients was maintained during the study, and all information provided by the patients was treated with utmost confidentiality. Patients' consents were sought and formally obtained after a detailed explanation of the intention of the author concerning the research findings. Ethical approval for this study was sought and received from the Ethical Committee of the University of Calabar Teaching Hospital.

## 3. Results

The Age distribution of the respondents varied with the highest proportion being young adults aged 26–40 years (44%), adolescents aged 18–25 years (34%), middle-aged persons aged 41–60 years (18%), and elderly patients aged 60 + 9 (4%), Table 1. Two hundred and fifty-five (59.3%) were satisfied with their patient-doctor encounter.

The average age of the respondents was 29 years while the average age of all the patients who presented to the hospital during the study period was 31 years.

The sex ratio was almost equal with males accounting for 201 (47%) and females 229 (53%) of the respondents (Table 1). There was a wide variation among the occupational characteristics with patients who had any form of paid employment accounting for 27%, students 32%, retired persons 5%, unemployed 9%, housewives 7%, and others 22% (Table 1). Among the others were artisans, self-employed businessmen, and farmers. Sex and occupational distribution of the respondents were shown to be similar to those of all the patients who presented in the clinic during the study period. 

Majority of the patients 230 (53%) felt the time they spent with the physician was adequate or very adequate (Table 2). Only 26 (6%) respondents assessed the time they spent with the physician as inadequate (Table 2). Two hundred and twenty-five (52%) respondents felt they understood the illness much more than when they came to visit the doctor (Table 2). 

Seventy-eight percent of the patients who participated in the study and perceived that the encounter had made it possible to cope with the illness were satisfied with their encounter (Table 2). A good majority of the patients perceived that their ability to cope with the illness after the visit influenced the patients' satisfaction with the encounter (*P* < 0.001). 

 Three hundred and fifty (81%) of patients who found an improvement in their ability to maintain their health were satisfied (Table 2) with their encounter (*P* < 0.001). 

Table 3 shows that the frequency of visits did not statistically influence the patients' satisfaction with the consultation (*P* > 0.25). The patient's assessment of time spent in the consultation was shown to have a statistically significant influence on the patients satisfaction with the consultation (*P* < 0.001). This table also shows that the patients preference for a particular physician did not statistically influence the patients satisfaction with the consultation (*P* > 0.05). 

## 4. Discussion 

None of the sociodemographic variables studied were found to have any statistically significant relationship with a patient satisfaction in a consultation. This study could not demonstrate any statistical significance between a patient age and their satisfaction with the consultation. This agrees with some studies which demonstrated similar findings [8, 9] but differs from other studies which demonstrated that patients' satisfaction rates usually improve with advancing age [10, 11]. 

The elderly patients included in this study were only 18, and perhaps with a larger elderly population the study could have demonstrated an age-related effect on satisfaction in the consultation. Many elderly patients did not agree to complete their questionnaires, and this was probably due to the influence of the accompanying persons who often insisted they had to return to work as soon as possible. 

The living arrangements in our society possibly make us share similar illness perceptions of what is good or bad accompanied by a shared cultural understanding of wellness or illness. 

Sex may influence certain conducts in a consultation. The sex distribution was almost equal. This study could not demonstrate any statistically significant influence of a patient's sex on his/her satisfaction with a medical encounter (Table 1). Forty-seven percent of patients were male, while 53.26% were females. 

The patients' frequency of visits to the GOPC was not found to statistically influence the patient's satisfaction with the consultation (Table 3). It was believed that the higher the number of visits, the higher the level of dissatisfaction because this was thought to be related to the higher likelihood of social factors not being addressed in these frequent users of the hospital services. However, the findings suggest otherwise. 

Patients often request to see particular doctors, but this was not shown to influence their satisfaction in this study (Table 3) and is supported by a study among Israeli patients [12]. This finding differs from other studies which have shown that continuity and being seen by a particular doctor improve concordance and satisfaction [10, 13, 14]. 

The difference in this study may be accounted for by the fact that the patients were in a teaching hospital and many were usually referred to other clinics when the need arose. Many patients expect this as they come and may be psychologically prepared. This may explain the fact that 95% of patients in this study did not insist on seeing a particular doctor as many patients often see the clinic as a transit route to other specialist clinics. Only about 32% of patients had been to the clinic on at least three previous visits with the majority (44%) having attended just 1-2 times or with no previous visits (25%) in the last 12 months. It is possible they had not cumulatively spent enough time with the doctors to form an opinion. There is also the practice of doctors changing rooms, duties, and postings in between patients' visits. This makes patients wary of requesting for a particular doctor who may not be on duty. Usually in the study area clinic, patients are not given the choice of selecting a doctor and may be rebuked by the nurses who do the sorting if they request for a particular doctor. Also patients probably did not request for a particular doctor because they did not know the doctors, were not aware if a particular doctor was on duty, or how long they needed to wait to see a preferred doctor. 

The use of time in the consultation has been shown to be crucial to consultation satisfaction ratings [13]. This study demonstrates a statistical significance between patients' perception of time spent in the consultation and satisfaction (Table 2), but not all studies agree [12]. Fifty-three percent of the patients rated the time spent in their consultation as adequate or very adequate, with 47% describing the time spent as either fair or inadequate (Table 2). 

Patients' assessment of the adequacy of time is crucial in gauging their satisfaction as it has been linked to satisfaction with psychosocial issues in the consultation. Patients' assessment of time spent in the consultation may be influenced by certain individual traits such as age [15]. In this study, patients often confused the time spent in the consultation with the time spent in the waiting room, and throughout the data collection patients were encouraged to make this distinction as they completed their questionnaires. The patient perception of time is crucial in the consultation, and this influenced whether the patient was satisfied with the consultation or not. 

Duration of a patient illness has been shown to have an influence on the consultation by a study of chronic illnesses while a patient satisfaction with a consultation can also affect the duration of his illness [16]. However, this study could not demonstrate any statistically significant influence of chronic illness on patients' satisfaction (Table 3). 

Patients with chronic illnesses are expected to know more about their illness than those with acute illness and are thought to require more attention. Chronic illnesses are usually not what doctors expect to manage when they graduate, and their management may be a form of psychological burden to the physician [17]. This study did not demonstrate any effect on satisfaction rates by the presence or absence of a chronic illness (Table 3). This finding may be explained by the fact that many patients in our environment are not well informed about their illnesses, so their knowledge of the illnesses does not necessarily increase as the durations of their illnesses increase. 

This study could not demonstrate any significant influence of occupation on a patient's satisfaction with the consultation (Table 1). Students made up 32% of the respondents, employed persons 27%, and housewives 7%. Despite this spread, no difference was demonstrated. 

The patient's occupational status is closely linked to the person paying for their medical expenses. This was also found not to have any statistically significant influence on patients' satisfaction with their consultation (Table 1). Fifty-two percent of the patients were paying for their medical expenses themselves while families were paying for 30%. Ten percent of the patients including two males were being paid for by their spouses. However, 3% of the patients did not know who would pay and an equal number was being sponsored by their employers. 

The number being sponsored by their employers was unexpectedly low (2.6%) considering the Nigerian Government's efforts at promoting a National Health Insurance Scheme (Table 1). The effect of managed care in this study cannot be discussed owing to the low number of patients who were using health care insurance, but studies in the United states have demonstrated that managed care affects neither the perception of time used in the consultation [16] nor patients' satisfaction with it [17]. There was also a possibility that the number of students might be lower than the observed figures because some young people in Calabar town often claimed to be students when they were not. This finding suggests that people from different occupational backgrounds in our practice environment may not bring their psychological expectations to influence their satisfaction with the consultation. 

The more a consultation contributes to a patient's understanding of his illness, the higher the likelihood for the patient to be satisfied with the consultation [18, 19] but very often patients get less information than they expect [20]. Fifty-nine percent of the patients had a satisfying consultation, while 52% had some improvement in the understanding of their illness. However, of the 41% of patients with unsatisfying consultations, 28% of them still had an improvement in their illness understanding (Table 2). This suggests that, despite the lack of illness understanding there is still some satisfaction with the consultation. However this study clearly demonstrates that a patient understanding of his/her illness has a statistically significant effect on the patient's satisfaction with the consultation (Table 2). It seems clear that the more informing a consultation is, the more likely a patient is to be satisfied with the consultation [21]. This finding supports the call by one report [22] for the patient to be more involved in decision making. Many patients were observed in this study to have shown great interest when they found the doctor to be willing to provide some explanation about their illness. 

A patient's ability to cope with his illness can be helped or marred by a consultation, and this is more evident in chronic illnesses [23]. Patients' abilities to cope with their illness based on the information received have been demonstrated by this study to statistically affect patients' satisfaction with the consultation (Table 2). It is increasingly clear that better informed patients have better outcomes, choose less risky procedures, and avoid equivocal treatments [11]. 

A patient's ability to maintain health after a consultation would be addressing one of the core issues in family medicine by promoting prevention of illnesses. This study demonstrates a statistically significant effect of a patient's ability to cope with his illness on his/her satisfaction with the consultation (Table 2). This is vital in our environment considering that the bulk of illnesses we manage is due to preventable diseases. 

In conclusion, client satisfaction with patient-doctor encounter is high. Factors influencing the patient-doctor consultation are numerous, and the exact influence of any of these factors is not easily isolated, but together these factors influence the interaction either positively or negatively. However, the study has shown that, despite the various factors that are considered to encourage client satisfaction at primary care consultation, a few of such factors contributed to end of consultation satisfaction in our environment. This calls for a refocusing if improvement in the overall patient care in our cultural context is to be achieved with the aim of meeting patient needs. Teaching of consultation process must take these factors into consideration. 

To further address effort towards improving patient satisfaction rates in the study centre, it is recommended that the physician-related factors that influence the doctor-patient encounter should be further studied. In particular the effect of sociodemographic variables such as same-sex consultation, cultural/language diversity, and experience should also be further explored. 

## Figures and Tables

**Table 1:** Demographic characteristics of the study subjects, *N* = 430.

Categories	Satisfaction with the consultation		Total	*χ* ^2^	df	*P* value
	Yes	No				
	*N* (%)	*N* (%)	*N* (%)			
Age in year						
<25	86 (20.0)	60 (14.0)	146 (34.0)	4.370	3	0.5
26–40	118 (27.4)	71 (16.5)	189 (44.0)			
41–60	43 (10.0)	34 (8.0)	77 (18.0)			
>60	8 (1.9)	10 (2.3)	18 (4.2)			
Sex						
Male	119 (27.7)	82 (19.1)	201 (46.7)	0.0013	1	0.5
Female	136 (31.6)	93 (21.6)	229 (53.1)			
Occupation						
Employed	71 (16.5)	44 (10.2)	115 (26.7)	1.7125	6	0.5
Unemployed	24 (5.6)	15 (3.5)	39 (9.1)			
Student	77 (17.9)	60 (14.0)	127 (31.9)			
Unable to work	11 (2.6)	5 (1.2)	16 (3.7)			
Housewife	18 (4.2)	12 (2.8)	30 (7.0)			
Retired	12 (2.6)	10 (2.3)	22 (5.2)			
Others	42 (9.8)	29 (6.8)	71 (16.5)			
Source of funding						
Spouse	24 (5.6)	18 (4.2)	42 (9.8)	1.636	5	0.5
Self	134 (31.2)	90 (20.9)	224 (52.1)			
Family	78 (18.1)	49 (11.4)	127 (29.5)			
Friends	7 (1.6)	6 (1.4)	13 (3.0)			
Employer	6 (1.4)	5 (1.2)	11 (2.6)			
Do not know	6 (1.4)	7 (1.6)	13 (3.0)			

**Table 2:** Patients' assessment of time with the physician, illness understanding after the consultation, ability to cope with illness, and ability to maintain health after the visit.

Categories	Responses	Satisfaction with the consultation		Total	*χ* ^2^	df	*P* value
		Yes	No				
		*N* (%)	*N* (%)	*N* (%)			
Patient's assessment of the time spent in the consultation	Inadequate	6 (1.4)	20 (4.6)	26 (6.1)	65.79	3	0.001
	Fair	75 (17.5)	99 (23.0)	174 (40.4)			
	Adequate	115 (26.7)	49 (11.4)	164 (38.1)			
	Very adequate	59 (13.7)	7 (1.6)	66 (15.4)			

Illness understanding after visit	Much more than before the visit	155 (36.1)	68 (15.8)	223 (51.9)	30.17	3	0.001
	Little more than before the visit	70 (16.3)	52 (12.1)	22 (28.4)			
	Same or less than before the visit	6 (1.4)	10 (2.3)	16 (3.8)			
	No reply	24 (5.6)	45 (10.5)	69 (16.1)			

Ability to cope with illness after visit	Much more than before the visit	148 (34.4)	52 (12.1)	200 (46.5)	34.52		0.001
	Little more than before the visit	67 (15.6)	69 (16.1)	136 (31.6)			
	Same or less than before the visit	14 (3.26)	19 (4.4)	33 (7.7)			
	No reply	26 (6.05)	35 (8.14)	61 (14.2)			

Health maintenance after visit	Much more than before the visit	156 (36.3)	73 (17)	229 (53.3)	20.16	3	0.001
	Little more than before the visit	66 (15.3)	55 (12.8)	121 (28.1)			
	Same or less than before the visit	10 (2.3)	10 (2.3)	20 (4.7)			
	No reply	23 (5.4)	37 (8.6)	60 (14.0)			

**Table 3:** Frequency of patients visits to the GOPC, willingness to be consulted by any physician during the visit, and presence or absence of any long-standing illness.

Categories	Responses	Satisfaction with the consultation		Total	*χ* ^2^	df	*P* value
		Yes	No				
		*N* (%)	*N* (%)	*N* (%)			
		*N* = 255	*N* = 175	*N* = 430			
Frequency of patients' visit to the General outpatient clinic over the previous 12 months	None	62 (14.4)	45 (10.5)	107 (24.9)	3.68	4	0.25
	1-2	115 (26.7)	73 (17.0)	188 (43.7)			
	3-4	55 (12.8)	38 (8.8)	93 (21.6)			
	5-6	11 (2.6)	14 (3.3)	25 (5.8)			
	≥7	12 (2.8)	5 (1.2)	17 (4.0)			

Willingness to be consulted by any physician during the visit	Yes	241 (56.1)	167 (38.8)	408 (94.9)	3.61	1	0.05
	No	14 (3.3)	8 (1.9)	22 (5.1)			

Presence or absence of any long-standing illness	Yes	58 (13.5)	37 (8.6)	95 (22.1)	0.5	1	0.5
	No	197 (45.81)	138 (32.90)	335 (77.90)			
